# Viral Bronchiolo-Alveolitis From Coronavirus OC43 and Rhinovirus-Simulating SARS-CoV-2 Infection

**DOI:** 10.7759/cureus.28093

**Published:** 2022-08-17

**Authors:** Fatima Zahra Alaoui-Inboui, Bouchra Slaoui

**Affiliations:** 1 Pediatrics and Child Health, Hopital D'enfant Abderrahim Harouchi, Casablanca, MAR; 2 Service de Pneumo-allergologie, Hopital D'enfant Abderrahim Harouchi, Casablanca, MAR

**Keywords:** coronavirus oc43, infant, life-threatening complications, multiplex pcr, pneumomediastin, respiratory tropism viruses, rhinovirus, sars-cov2 infection, severe evolution, viral bronchiolo-alveolitisum

## Abstract

Viruses are the most common cause of acute lower respiratory tract. The respiratory syncytial virus infection is most commonly associated with viral bronchiolitis. Rhinovirus and coronavirus OC43 are less frequently responsible for lower respiratory tract infections. The virological spectrum has expanded greatly owing to the development of molecular biology techniques. We report the clinical case of a four-month-old infant who presented with acute lower respiratory infection with coronavirus OC43 (HCoV-OC43) and rhinovirus complicated by a particularly extensive spontaneous pneumomediastinum.

## Introduction

Viral bronchiolitis represents a very frequent and potentially serious form of viral respiratory infections in children. Respiratory syncytial virus (RSV) infection is the most common cause of viral bronchiolitis. However, a number of other respiratory viruses have been isolated in viral bronchiolitis, among the traditional viruses: influenza viruses, para-influenza viruses (PIV), adenoviruses, some rhinoviruses and the “new” respiratory viruses: human metapneumovirus (hMPV), and bocaviruses [1]. Other than COVID-19, coronaviruses are involved in 8% of viral bronchiolitis cases, mainly OC43, NL63, and HKU1 coronaviruses. The clinical presentation of OC43 corona infection may be difficult to differentiate from severe acute respiratory syndrome coronavirus 2 (SARS-CoV-2) infection. In this study, the molecular biology methods detected the viruses in a nasopharyngeal sample with a multiplex polymerase chain reaction.

## Case presentation

**Figure 1:**
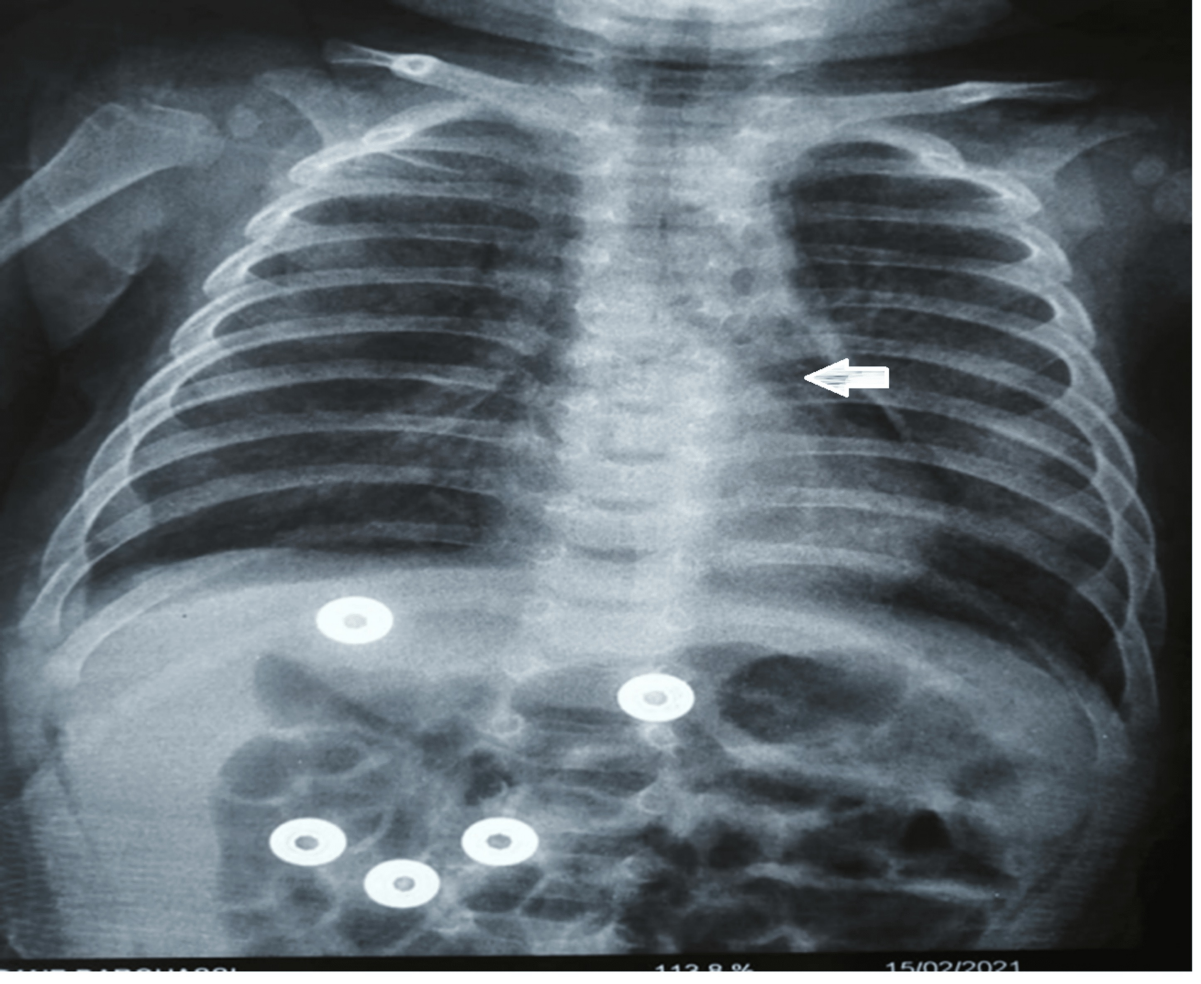
Thoracic distension with a clear gaseous border silhouetting the left edge of the mediastinum in relation to the pneumomediastinum. A clear gaseous border silhouetting the left edge of the mediastinum in relation to the pneumomediastinum

The case concerned a four-month-old infant, admitted on February 14, 2021, for acute viral bronchiolitis, without a past history of illness. Clinically, the patient had a dry then productive cough, intense signs of respiratory struggle caused by the retractions and expiratory braking with a polypnea at 64 breaths per minute. General examination revealed a temperature of 37.6°C and thoracic distension without wheezing during the check-up. Pulse oxygen saturation (SpO_2_) in ambient air was 94%. The biological check-up showed non-significant inflammatory syndrome (C-reactive protein at 15 mg/L and hemogram leucocytosis at 13.990/mm³, predominantly neutrophils at 9.860/mm³ and lymphocytes at 3.680/mm³). Symptomatic management was established as follows: 30° proclive position, oxygen therapy with oxygen goggles, and intravenous rehydration. The infant's clinical setting worsened 12 h after hospitalization. He developed severe respiratory distress consisting of perioral cyanosis, shallow breathing, expiratory dyspnea, increased signs of breathing struggle with tachypnea at 75 CPM, and increased oxygen requirements (SpO_2_ to 83% in the ambient air). Chest x-ray showed thoracic distension associated with a clear gaseous border silhouetting the left edge of the mediastinum related to pneumomediastinum (Figure 1).

**Figure 2:**
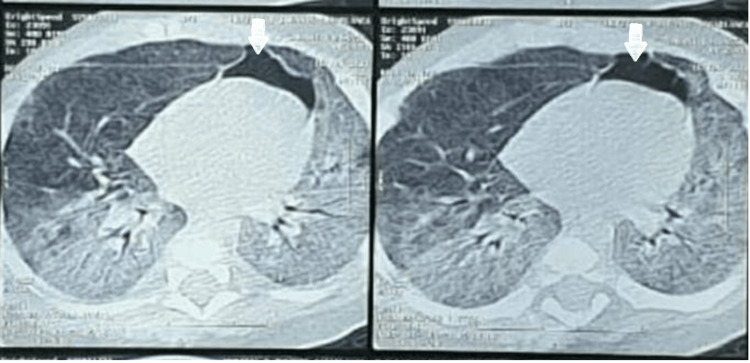
Diffuse frosted glass surfaces, more bilateral at the lower lobe (left and right), suggesting diffuse interstitial pulmonary disease looking infectious and a pneumomediastinum of average abundance A pneumomediastinum of average abundance

The chest x-ray was completed by a thoracic scan which revealed diffuse ground-glass opacity surfaces, more bilateral at the lower lobe, suggesting diffuse interstitial pulmonary disease looking infectious and a pneumomediastinum of average abundance (Figure 2).

During the current COVID-19 pandemic, the biological assessment was enhanced by PCR using SARS-CoV-2 and multiplex PCR. The polymerase chain reaction (PCR) of SARS-CoV-2 and COVID-19 serology (anti-SARS-CoV-2 IgG antibodies) were negative. Multiplex PCR highlighted coronavirus OC43 and human rhinovirus. The patient was transferred to the pediatric intensive care department for further management. In the intensive care unit, and due to the severity of the initial clinical picture, the patient was put on antibiotics without waiting for the results of the PCR. He was administered a third-generation antibiotic therapy based on cephalosporin at a rate of 50 mg/kg/d with intravenous corticosteroid therapy (Methylprednisolone 2 mg/kg/6 h). The clinical course revealed a decrease in respiratory struggle and oxygen requirements: SpO_2_ = 98% (under 3 L/min of oxygen), Respiratory rate was 54 breaths per minute, and T =37 °C. A definitive oxygen withdrawal was possible 48 h later with complete regression of respiratory breathing struggle signs and improvement of the control chest x-ray. The patient was allowed back home on the 12th day.

## Discussion

This is a case report of a four-month-old infant who presented with severe bronchiolo-alveolitis due to coronavirus OC43 and human rhinovirus complicated by a spontaneous pneumomediastinum with a favorable outcome. Respiratory tropism viruses vary and are often responsible for lower respiratory tract infections. In such cases, they cause bronchiolitis, bronchitis, or pneumonia, mainly in newborns and young infants. New virological diagnostic tools allow for the refinement of epidemiological data. PCR techniques highlight a much broader viral spectrum, including already known organisms and so-called “emerging” viruses that often result from genetic recombination. The emergence of coronavirus in the human population has led to a strong revival of interest and intensification of research on these viruses [1].

Among these viruses, RSV is found in 64.1% of acute bronchiolitis cases in hospitalized infants, human metapneumovirus (hMPV) in 7.6%, and parainfluenza virus (PIV) in 3.4% of cases. Respiratory infections may also be due to viruses not commonly acquired in practice, such as OC43 or 229E coronaviruses, or to newly identified viruses using molecular screening techniques, such as bocavirus or coronavirus NL63 and HKU1 [2].

Coronaviruses are a family of viruses that cause benign respiratory infections [3]. There were different distributions of acute community bronchiolitis: RSV (42%), rhinovirus-enterovirus (9.5%), coronavirus (8%), parainfluenza virus (3.5%), and hMPV (2.5%). RSV infects infants (86.9%), with 53.7% being less than 6 months old, 48.2% of RSV infections are detected in November and December, and 44.5% in January and February [4]. They have a higher seasonal incidence in winter and early spring. Coronaviruses are found in approximately 10 % of the samples of respiratory infections but are also responsible for lower infections with pneumopathy that may be serious in infants [5]. Coronavirus OC43, a coronavirus other than SARS-CoV-2, identified in 1965, is a hyper-endemic virus that affects young children. According to a study conducted in Caen between September 2004 and May 2005 on hospitalized children [6], 27 cases of viral respiratory infection with coronavirus OC43 were detected of which 63%of these infections were found in infants. Coronavirus OC43 causes upper and lower respiratory tract infections, including bronchitis and bronchiolitis, mainly in infants [7,8]. Occasionally, it can cause viral pneumonia in these patients. In a recent study in Hong Kong, viral bronchiolitis and pneumonia were diagnosed in 29.5% and 11.1% of NL63 coronavirus infections and 16.6% and 12.5% of OC43 coronavirus infections, respectively [9]. Simultaneous detection of various viral pathogens is frequently reported in children with respiratory infections (10%-40% of cases) [10].

In our case report, the patient had a viral co-infection with coronavirus OC43 and human rhinovirus, which was probably responsible for the severity of his respiratory infection. Rhinoviruses can cause bronchitis, bronchiolitis, or pneumonia in 5-40% of infants [11]. In rhinovirus infections, many radiological pulmonary abnormalities are associated with low respiratory concerns. Conversely, there is no atelectasis, obliterating bronchiolitis, or lesional edema as seen in RSV, measles, or adenovirus infections [12]. In a prospective study conducted in Spain over 14 years, Calvo et al. found that in two-thirds of cases, these respiratory infections were due to co-infection with other viruses. Pneumonia is more common in co-infections with coronavirus OC43 [13]. Other authors also found that children infected with HCoV-OC43 are often young, less than one year old [14]. There is concern about the clinical presentation of coronavirus OC43 in this immunocompetent young infant with a COVID-19 simulated presentation. Coronavirus OC43 infection is easily transmissible, without specific treatment, and could easily be mistaken for COVID-19 infection [15].

On the thoracic scan, there was mainly an uneven distribution of airspace, often at the lung periphery, thickening of the bronchial periphery, and frosted glass opacities, which is consistent with this case report. The present case of spontaneous pneumomediastinum is a relatively rare complication of more severe viral bronchiolitis, regardless of the virus. This underlines the importance of careful management and the use of chest computed tomography for a follow-up to ensure no delay in diagnosing complications associated with viral bronchiolitis [11].

## Conclusions

Severe viral respiratory infections can lead to life-threatening complications. This study shows that the HCoV-OC43 strain may be clinically more significant than previously thought since it is easily communicable, can cause respiratory failure, and potentially has important public health significance, also the rhinoviruses can cause severe lower respiratory infections in infants. The rhinovirus - coronavirus OC43 co-infection can thus generate a severe viral infection as shown by this reported case. Gene amplification by RT-PCR greatly increases virus detection, thus promoting respiratory virus documentation to rationalize the use of antibiotics.
